# Dr. Charles Glidden

**Published:** 1918-06

**Authors:** 


**Dr. Charles Glidden** of Dansville died suddenly March 24th. He was a member of the medical staff of the Jackson Health Resort until the government rented the institution for a base hospital. At the time of his death he was in charge of the patients of this sanitarium as they had been removed to the Frederick Noyes home in the village while the Avon Inn at Avon, N. Y., is being repaired for their occupancy.

